# Closing the Gaps to Timely Patient Access: Perspectives on Conditional Funding Models

**DOI:** 10.3390/curroncol29020083

**Published:** 2022-02-10

**Authors:** Judith Glennie, Eva Villalba, Paul Wheatley-Price

**Affiliations:** 1 J.L. Glennie Consulting Inc., Aurora, ON L4G 7G5, Canada; 2 Quebec Cancer Coalition, Saint-Lambert, QC J4P 2J7, Canada; evav@coalitioncancer.com; 3 Ottawa Hospital Research Institute, The Department of Medicine, University of Ottawa, Lung Cancer Canada, Ottawa, ON K1H 8L6, Canada; pwheatleyprice@toh.ca

**Keywords:** health technology assessment, medication access, conditional reimbursement, unmet medical need, patient outcomes

## Abstract

The Canadian system for approval of new cancer drugs is complex with multiple steps. Health Canada grants a license for a drug to be marketed and prescribed. The Canadian Agency for Drugs and Technologies in Health (CADTH) and Institut national d’excellence en santé et services sociaux (INESSS) make recommendations by way of health technology assessments (HTA). If positive, the latter then lead to confidential price negotiations at the pan-Canadian pharmaceutical alliance (pCPA), after which individual provinces and territories make a listing decision. Delays can occur at each stage, but post-HTA delays can be lengthy and unpredictable, denying or impeding access to an effective drug with the potential for devastating clinical outcomes. Conditional funding models have been adopted in a number of European countries with the goal of providing timely access to new medications in areas of unmet need, in advance of further steps in the reimbursement process. This manuscript discusses different stakeholder perspectives on conditional funding agreements—including a recent successful example of such a process in the UK—based on a panel discussion at the 2021 Canadian Association of Population Therapeutics (CAPT) Conference.

## 1. Introduction

The following summary is based on a panel session delivered at the Canadian Association of Population Therapeutics (CAPT) Conference on 25 October 2021 and is derived from information and insights shared by the presenters. CAPT (https://www.capt-actp.ca/; accessed on 30 December 2021) is a not-for-profit member-driven organization whose participants include scientists and researchers, government, industry and consulting, academia, and the patient community. Conference topics typically include the disciplines of epidemiology, economics, and policy decision making and beyond; and, the overall 2021 meeting theme was “Evidence in a Time of Crisis”.

The session’s objective was to discuss how stakeholders can work together to create a system that enables timelier patient access to medications funded by public drug plans, with a specific focus on how a conditional listing mechanism might be a useful tool in promoting patient access in certain instances. The panel members provided patient, clinician, and industry perspectives on real-world examples and/or the potential value of such a mechanism in the Canadian environment. 

## 2. Background

In terms of finding ways to improve timely patient access to medications via public drug programs, Health Canada, the Canadian Agency for Drugs and Technologies in Health (CADTH), and Institut national d’excellence en santé et services sociaux (INESSS) have put significant effort into accelerating regulatory and health technology assessment (HTA) reviews for products where early clinical data are promising and there is high patient need. Under Project Orbis [1], an international regulatory partnership has been forged between the US, UK, Canada, and several other countries to give cancer patients faster access to promising cancer treatments. Within the Canadian environment, this accelerated regulatory approach then enables Health Canada and HTA bodies to move forward on the aligned reviews they have developed over the past few years [2]. 

Unfortunately, these advances have been undermined by later stages of the reimbursement process, where delays in negotiations at the pan-Canadian Pharmaceutical Alliance (pCPA) and/or the provincial listing have become more the norm than the exception [3]. Negotiation delays can be caused by a variety of factors. For instance, delays occur in the time for pCPA to pick up a file for negotiation after the HTA review process is complete. Once negotiations are initiated by pCPA, the time to complete the negotiation and come to an agreement is influenced by both pCPA and the pharmaceutical company involved. Regardless of reason, patients are waiting extended periods of time until public payer funding of new medications is available. While company-sponsored patient support programs (PSPs) have often filled the funding gap for patients for many years, they are becoming less sustainable with the lengthening time to provincial listings; and, have wide variability in their access depending on the drug, the indication, the jurisdiction, or the company involved. PSPs can, therefore, mask significant and problematic delays in the system, which is ultimately not meeting patient needs and can create inequities in access.

**Figure 1:**
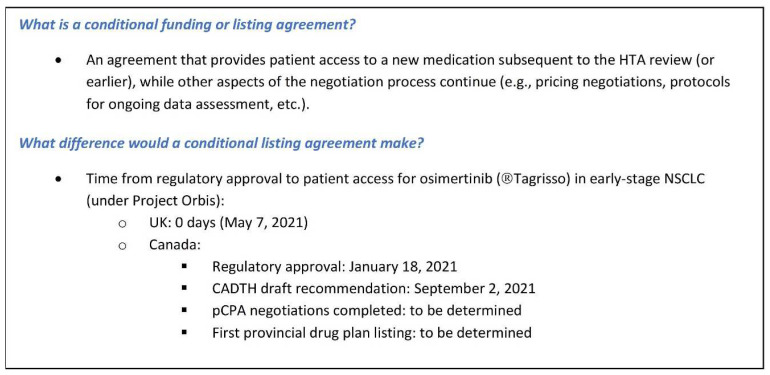
Conditional listing agreement—definition and potential impact.

What is needed to overcome these challenges is for all parties to demonstrate a willingness to be creative and work together to develop options for accelerating access to medicines in areas of high unmet medical need. A conditional funding mechanism could significantly decrease the delay between regulatory approval and patient access for new medications that demonstrate a significant clinical benefit and meet an important unmet need (see Figure 1; also, further information later in the paper).

Similar to the osimertinib example in Figure 1, the UK’s National Health Service (NHS) moved forward with an accelerated patient access for sotorasib, a breakthrough KRAS inhibitor for the KRAS G12C non-small cell lung cancer (NSCLC) subtype. The conditional listing in this case involves a budget neutral agreement that allows the drug to be used very soon after market authorization, in return for a substantial pricing discount and a commitment to real world evidence (RWE) generation and to support a future HTA evaluation [4]. Other countries have similarly acknowledged the need to address administrative process delays that are impacting patient access. For instance, France recently introduced proposed measures under their Health Innovation 2030 Plan [5] to accelerate market access to innovative medicines. Under the proposed system, there will be immediate market access to all products that receive an “Improvement in Actual Medical Benefit” (IAMB/ASMR) rating of I to IV under the French HTA process, with further on-going data collection, evaluation, and/or price negotiations thereafter. This is similar to the approach that has been in place in Germany for some time.

Clearly, other jurisdictions are looking at approaching patient access differently, in response to the introduction of important new medications. There is a great deal that Canada can learn from other countries, regardless of differences in our health and reimbursement systems.

The following summary is intended to advance discussions on creating a conditional funding framework or mechanism to enable more timely access to innovative new medication—based on the UK experience and integrating both patient and clinician perspectives.

## 3. The UK Experience

The osimertinib accelerated access example previously noted (see Figure 1) was the result of close collaboration across multiple government agencies in the UK—the Medicines and Healthcare products Regulatory Agency (MHRA), the National Institute for Health and Care Excellence (NICE), and National Health Service (NHS) England—in partnership with the sponsoring company, AstraZeneca. This collaboration ultimately established a new mechanism for patient access which is now open and available for other innovative cancer medicines coming to market in the UK.

In Spring 2020, the data readout for the osimertinib ADAURA study resulted in the trial being stopped two years early due to overwhelming efficacy. The study investigated osimertinib versus placebo as an adjuvant therapy after the resection of EGFR-positive NSCLC, and demonstrated an approximate 80% reduction in the risk of recurrence in the cohort receiving osimertinib [6]. In turn, the therapy was given breakthrough status by the FDA and integrated into the Project Orbis process. The UK’s MHRA joined Project Orbis as an observer for the purposes of this file, which enabled them to make a faster decision on marketing authorization for patients in the UK in early May 2021. In addition to MHRA’s actions, NICE also developed plans to accelerate their appraisal timelines nine months ahead of schedule in partnership with the company. This reflected not only the important data generated in the study, but also NICE’s commitment to reducing the gap between marketing authorization and reimbursement.

In addition to MHRA and NICE’s actions to accelerate the time to patient access, there was still one remaining challenge in closing the gap between market authorization and funding. Drug funding in the UK is predicated on a positive outcome in the NICE appraisal. AstraZeneca engaged in discussions with NHS England, which ultimately resulted in the creation of a mechanism to bridge the period of time between the marketing authorization granted by the MHRA while the NICE appraisal process continued. Of note, as one of the conditions of the risk sharing agreement in place with NHS England, AstraZeneca has committed to funding treatment for the duration of the care that any patient who has access to osimertinib under the contingent approval or interim funding mechanism needs.

In terms of using this new system between NICE, NHS England, and pharmaceutical manufacturers for future medications, the following criteria have been developed:

- A qualifying medicine must hold an innovation passport (i.e., be identified as a new innovation in an area of unmet need) or designated via an accelerated regulatory pathway such as Project Orbis.
- The system also needs to confirm that there is an unmet need (NICE and NHS England).
- The company must be ready to make a submission (i.e., HTA evidence dossier ready to go).
- Negotiations to ensure that the risk is shared between the company and between NHS England during that period when the contingent approval is made available.

The key lesson learned from this example is that it is possible to bring multiple agencies and stakeholders together to work towards a common goal of supporting patient access to important new medications that have an impact on unmet medical needs. On-going communication and collaboration amongst all parties were also key success factors, along with the willingness to do things differently in order to address patient needs.

## 4. Patient Perspective

As a leading patient group in the province, the Quebec Cancer Coalition is promoting the need for modernization of the HTA and overall reimbursement process. The current standard of evidence based on randomized controlled trials with a control arm, etc., reflects yesterday’s innovations, while precision medicine is the future. Patient groups are concerned that current HTA approaches are not aligned with the modern era and are linked to concerns regarding timely patient access, especially in light of the paradigm shift required for precision medicine.

**Figure 2:**
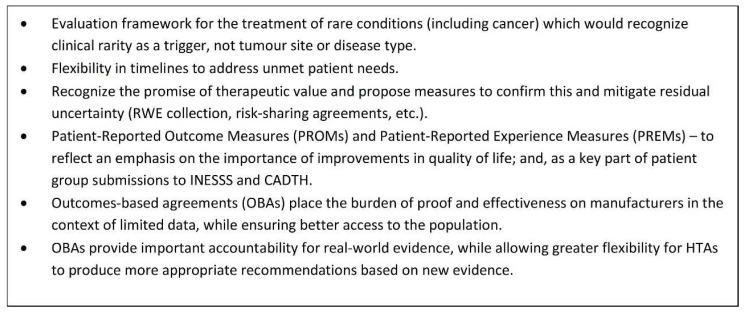
Proposal to INESSS: Framework for rare conditions, conditional approvals, OBAs and RWE [7].

To address these concerns, the Cancer Coalition has submitted a proposal to INESSS in an effort to influence HTA processes in Quebec and the rest of Canada. Figure 2 outlines the key issues outlined in the proposal and the direction that the Coalition would like to see HTA move in the near future. In particular, outcomes-based agreements (OBAs) are seen as a very effective tool for improving access because they place the burden of demonstrating real-world effectiveness (RWE) on manufacturers in the context of limited data while at the same time providing patient access.

**Figure 3:**
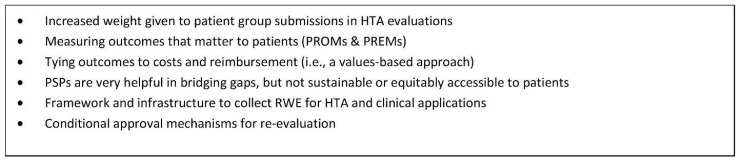
What is missing for improved patient access?

**Figure 4:**
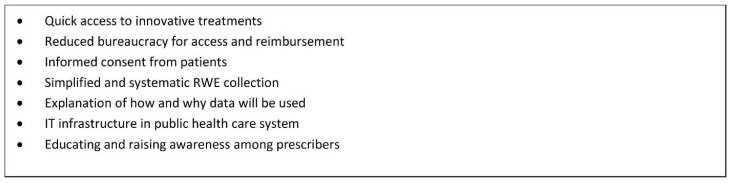
Best case scenario for a conditional listing mechanism.

A conditional funding approach to access provides a much better alternative than no access at all, which is what patients are currently experiencing as a result of the significant delays in various parts of the reimbursement process. The integration of RWE generation under an OBA provides the flexibility for HTA bodies to provide recommendations that can be contingent on new evidence and also provides important information that is relevant to the clinical setting. Figure 3 provides an outline of actions required to achieve improved patient access, which includes a conditional approval or listing mechanism. Figure 4 provides a summary of considerations for a conditional listing mechanism from a patient perspective.

## 5. Clinician Perspective

As stakeholders within the broader medical and medication access system, clinicians want effective medications to be available to their patients and are motivated to have access to those new products as quickly as possible. As noted earlier, a conditional funding mechanism would provide a potential solution to the delays and lack of transparency associated with the current pCPA process. This is particularly true for innovative new medications whose reviews have been accelerated by the regulatory and HTA parts of the reimbursement process. 

Within the last 2 years, there have been several very progressive HTA recommendations that have recognized the value of treatments, particularly in rare subpopulations of patients with unique data sets (e.g., crizotinib [8] for ROS1 NSCLC, dabrafenib/trametinib [9] for BRAF-positive NSCLC, larotrectinib [10] as a tumor agnostic treatment [NTRK fusion cancers]). Historically, CADTH reimbursement reviews (formerly the pan-Canadian Oncology Drug Review [pCODR]) required randomized clinical trial data to make a positive recommendation, but these latest examples represent approvals in rare cancers with compelling non-randomized or phase II clinical trial data. In the case of these products, the time from HTA recommendation to the completion of pCPA negotiations has been lengthy and unpredictable—10 months in the case of crizotinib, 5 months for larotrectinib, and still in progress for dabrafenib/trametinib (HTA recommendation 28 May 2021; initiation of negotiation 9 October 2021).

While PSP are helpful, dependance on them for access to medication while waiting for administrative processes to conclude is neither sustainable nor sufficiently reliable to ensure equitable and consistent access for patients. Ultimately, a conditional funding mechanism would help to avoid what have become very real gaps between PSPs and government funding, where patients cannot get access to a treatment and cannot afford to pay for these treatments themselves. Patients can literally succumb to their illness because of the gaps caused by these administrative processes.

**Figure 5:**
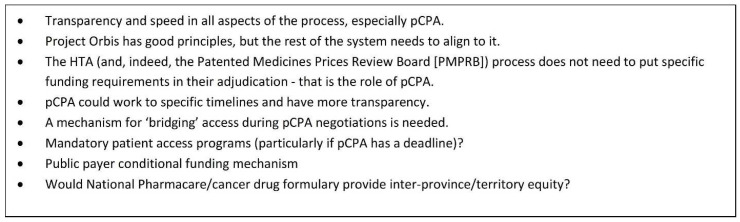
Suggested improvements to the reimbursement process. Discussion and Potential Limitations.

Figure 5 outlines some key improvements to the reimbursement process which should be considered in order to accelerate patient access to important new medications. A key recommendation would be to move forward in the development of a conditional funding mechanism that would come into play immediately after a CADTH recommendation in order to close the current access gap. Doing so would alleviate significant stressors for clinicians and patients: the moral distress that exists for clinicians knowing that an effective treatment is “available” but “not available”, and the very real physical and emotional distress and threat to health and life that exists for those patients who have to wait for lengthy administrative processes to be completed. Policies outlining such an approach would clearly need to assess the risk of such a system to the ultimate pricing negotiation (which the British, French, and Germans appear to have worked through) and include a commitment to patients if the negotiation and/or listing is not completed.

## 6. Discussion and Potential Limitations

Conditional reimbursement mechanisms are intended for products that demonstrate a significant clinical benefit and meet an important unmet need—with a particular focus on potentially important treatments approved through accelerated regulatory access processes. The latter create challenges for payers, who are pressured to fund these products on the basis of less evidence and, thus, greater uncertainty in their value for money. Thus, payers actually need tools to address these uncertainties—from both evidence and pricing perspectives. Ignoring these challenges and avoiding the creation of new mechanisms to address them is not tenable—from either an ethical or a political perspective. 

Theoretically, the use of conditional reimbursement mechanisms to mitigate payer uncertainties could be interpreted as encouraging manufacturers to lower their commitment to evidence generation and lowering the bar overall for the evidence required to fund products. While a legitimate concern, the reality is that the degree of evidence generation required for a product is determined at an international level by regulators. A conditional funding mechanism—particularly one with a requirement for ongoing data generation in the real-world setting (such as the UK sotorasib example above) which feeds back into the HTA process—in fact raises the evidence generation bar for manufacturers. Towse and Fenwick have proposed a framework [11] that sets out conditions under which a conditional reimbursement mechanism can be of mutual benefit when perceptions of value for money and of the value of undertaking additional research differ between payers and innovators.

Some authors have suggested [12] that ending access to a product available under a conditional reimbursement scheme may be difficult. This is certainly an important consideration and is one of the many policy issues that should be addressed proactively as part of the overall conditional reimbursement agreement. For instance, as outlined in the osimertinib example above, it would be important to include a commitment to provide on-going funding for the treatment if the agreement lapses. Ultimately, it is important that risks to payers are mitigated under any conditional listing mechanisms that might be developed.

The Netherlands has had extensive experience with conditional reimbursement (accompanied by re-evaluation), particularly with orphan drugs for rare diseases [13]. A policy analysis of their experience notes the challenges of creating such mechanisms, from governance, implementation, and outcomes perspectives. There are many lessons to be learned from their experience, which was heavily based on an on-going dialogue and collaboration with stakeholders.

## 7. Conclusions

There are a number of international examples and proposals from patient groups and clinicians on how Canada could move forward with a conditional funding mechanism that would enable access to important new medications funded by public payers in a timely manner. There is an opportunity for Canadian decision makers to engage with leaders in these other countries and learn from these examples, to develop pilot projects that would inform a made-in-Canada approach to mitigating access delays due to lengthy administrative processes. 

A key success factor in moving forward will be the willingness of decision makers to embrace the need to collaborate with stakeholders and experiment with new models of patient access and finding new ways to manage uncertainty and risks. It will be key for any model developed to be patient-focused, to be developed in collaboration with a broad range of stakeholders, and to be capable of generating real-world evidence that can inform both patient care and policy.
